# Understanding climate change through the eyes of children: a qualitative study with secondary school students

**DOI:** 10.3389/fpsyg.2025.1670331

**Published:** 2025-12-17

**Authors:** Ümmühan Özcan Tan, Hilmi Demirkaya

**Affiliations:** 1 Türkiye Cumhuriyeti Millî Egitim Bakanligi, Ankara, Türkiye; 2 Department of Social Sciences Education, Faculty of Education, Akdeniz University, Antalya, Türkiye

**Keywords:** climate change, middle school students, qualitative research, environmental awareness, thematic analysis, student perceptions

## Abstract

This study examined secondary school students’ perceptions of climate change, their levels of interest, and their comprehension of the concept, as well as their views on its causes, perceived effects, and suggestions for enhancing sustainability education in schools. A qualitative methodology was employed to capture the students’ authentic perspectives and meaning-making processes. Semi-structured interviews were conducted with 37 students in grades 6 and 7 attending a public middle school in southern Turkey. Thematic analysis revealed that although most participants expressed concern and curiosity about environmental issues, their understanding of climate change was often fragmented or media-influenced by media sources. Students primarily associated climate change with observable environmental changes, such as rising temperatures and natural disasters, and less frequently with human activities or with policy implications. Notably, the participants emphasized the need for more engaging and practical learning experiences, including outdoor projects, environmental clubs, and media literacy activities within the school curriculum. These findings underscore the importance of enhancing climate literacy and sustainability education in schools, with a focus on integrating critical media awareness and participatory learning opportunities. The study concludes with recommendations for improving environmental education through student-centered and enquiry-based teaching methods.

## Introduction

Climate change represents one of the most pressing challenges of the twenty-first century, impacting not only the environment but also the social, economic, and educational domains (Hermans et al., 2024; Fortunato et al., 2025). As societies increasingly recognize the significance of sustainable living, educational systems hold a pivotal responsibility for cultivating environmental literacy and equipping young learners to understand and engage with global environmental challenges (Okada and Gray, 2023). Schools play a particularly influential role in shaping students’ environmental perspectives, values, and sense of empowerment (Murzyn et al., 2025). Understanding how children and adolescents perceive climate change is essential for developing effective educational strategies (Stevenson et al., 2018).

Students’ perspectives, misconceptions, and emotional responses are pivotal in shaping their engagement with sustainability issues and informing educators on how to enhance their comprehension (Idrissi, 2025; Strachan and Markwick, 2025). However, research often focuses on adult populations, frequently overlooking younger learners’ perspectives (Sprung, 2025). Examining these perspectives not only sheds light on how students interpret climate-related phenomena but also provides valuable insights into improving both classroom and experiential learning strategies (Mostacedo-Marasovic et al., 2025).

This study investigated middle school students’ perceptions, experiences, and educational needs related to climate change. Rather than assuming that young individuals can or should directly resolve the global climate crisis, this study explores their understanding of the issue and their recommendations for enhancing climate change and sustainability education in schools in Australia. These insights can aid educators and policymakers in developing more relevant, student-centered environmental education strategies that promote critical thinking, scientific literacy and media awareness. To achieve these objectives, this study addresses the following research questions:

*RQ1*: How interested are secondary school students in climate change and sustainability issues?

*RQ2*: How do secondary school students perceive and conceptualize climate change?

*RQ3*: How do students perceive the effects of climate change on their daily lives and local environment?

*RQ4*: What are their views regarding the causes and contributing factors of climate change?

*RQ5*: What suggestions do students propose for improving climate change and sustainability education in schools?

## Materials and methods

This section delineates the research framework, the participants involved, the instruments and methodologies employed for data collection, ethical considerations, data analysis, researcher self-reflection, and the measures implemented to ensure the study’s credibility and reliability.

### Research model

This study utilized a fundamental qualitative design rooted in an interpretivist epistemological framework, with the objective of understanding how individuals derive meaning from their experiences (Russo-Netzer and Davidov, 2025; Lim, 2024). This methodological approach was selected to examine secondary school students’ interests in, perceptions of, and understanding of climate change, as well as their views on its causes, impacts, and educational significance.

An inductive and qualitative methodology was used, emphasizing participants’ personal interpretations rather than validating pre-existing hypotheses. This approach facilitated the identification of emerging trends within students’ narratives and provided a comprehensive account of their understanding of climate change in educational contexts (Toogood, 2023; Cojocaru et al., 2025).

### Participants

This study employed both convenience and purposive sampling techniques (Banerjee et al., 2025). The sample comprised 37 students from Grades 6 to 7 at a public middle school in southern Turkey, which falls under the jurisdiction of the Turkish Ministry of National Education (MoNE). Of these participants, 23 were female (62.1 %), while 14 were male (37.9 %). Furthermore, 67.6% of the students, equivalent to 25 individuals, were in Grade 6, whereas the remaining 12 students, accounting for 32.4%, were in Grade 7.

The school was selected because of its convenient accessibility, as the researcher was employed there. This familiarity facilitated rapport and communication; however, it also raised potential concerns regarding the researchers’ influence. To mitigate any undue pressure, participation was entirely voluntary, with no academic, financial or material incentives provided. Students were assured that their decision to decline or withdraw from the study would not affect their academic standing.

To mitigate potential bias, the researchers abstained from directly recruiting participants. Instead, recruitment announcements were disseminated by an independent educator not associated with the research team. The final sample comprised only students who had obtained written parental consent and provided personal assent.

### Data collection tools

A semi-structured interview form was developed to examine students’ awareness, perceptions, and emotional responses to climate change (Idrissi, 2025). This instrument comprised demographic questions and nine open-ended questions addressing five domains: (1) interest in climate change, (2) sources of information, (3) conceptual understanding, (4) perceived impacts on life, and (5) recommendations for enhancing sustainability education.

The questions posed included the following: Are you up to date with the latest climate change developments? What are your primary sources of information? How would you characterize climate change? How does it affect your daily life? What types of educational activities or lessons enhance students’ comprehension of climate change? The validity of the content was verified through an expert review conducted by three specialists in environmental and social sciences.

### Data collection process

During the 2022–2023 academic year, data were collected through individual interviews conducted in private settings within the school to ensure participants’ comfort and confidentiality. The interviews were conducted in quiet classrooms or counseling rooms during regular school hours, with no peers or staff present.

To facilitate authentic expression, interviews were conducted in Turkish, the students’ native language. With their consent, all interviews were audio recorded and subsequently transcribed verbatim. To ensure both linguistic and conceptual accuracy, the excerpts included in this article were translated into English and then back-translated into Turkish by an independent bilingual researcher. Any discrepancies were collaboratively addressed and resolved to ensure semantic precision.

The interviews collectively spanned 542 min, with each participant contributing an average of approximately 15 min. After excluding incomplete responses, the analysis included data from 37 students. While the interviews provided valuable qualitative insights, the absence of additional sources, such as classroom observations or student work, is acknowledged as a methodological limitation. Future research should consider employing triangulation to enhance the depth and validity of analyses.

### Ethical principles

The research adhered to ethical standards for studies involving minors, ensuring that parental informed consent and student assent were obtained. Participation was entirely voluntary, with the option to withdraw at any time. Interviews were conducted privately to uphold autonomy and confidentiality, and the responses were anonymized using codes (S1–S37). No incentives were provided, and there were no psychological, social, or academic risks associated with the participation. To address potential power dynamics, the researcher assured the participants that their involvement or responses would not affect their grades or status. This study was approved by the Akdeniz University Social and Human Sciences Scientific Research and Publication Ethics Committee (Decision No. 66, dated 14 February 2023).

### Data analysis

Data were analyzed using inductive thematic analysis, following the six-phase model outlined by Braun and Clarke (2006): familiarization, coding, theme development, reviewing, defining, and reporting. The objective of the analysis was to identify recurring meanings and categories that emerged directly from the data rather than to test pre-existing theories. Coding was conducted manually and validated through peer debriefing with two independent, qualitative researchers. Any disagreements in theme categorization were resolved by consensus. The analysis was firmly grounded in the participants’ words, ensuring that the interpretations accurately represented authentic student perspectives.

### Researcher reflexivity

In the dual roles of Social Studies teacher and interviewer, the researcher emphasized reflexivity to ensure methodological clarity and rigor. During both the data collection and analysis phases, the researcher maintained reflexive field notes documenting assumptions, reactions, and interpretive decisions. To mitigate potential bias arising from prior familiarity with the participants, several strategies were employed: emphasizing the importance of voluntariness and confidentiality during recruitment, involving an independent teacher to assist with consent distribution, and soliciting input from external experts to review codes and emerging themes. These reflexive measures effectively balanced insider knowledge with analytical objectivity, thereby enhancing the credibility and dependability of the study.

### Credibility and trustworthiness

The credibility of the study was enhanced through prolonged engagement with the participants, peer discussions, expert evaluations, and the incorporation of direct quotations to underscore key themes. Although complete triangulation was not achieved, the integration of open-ended feedback, demographic data, and reflective documentation provided a comprehensive understanding of students’ perspectives. Dependability and confirmability were further strengthened by meticulous documentation of the coding procedures and reflective commentary.

## Results

The findings of this study are organized according to the sub-objectives. The results derived from the student interview forms were corroborated through tabulation. Data were collected from 37 students who participated in this study. The primary research question investigated middle school students’ interest in climate change by examining their news consumption patterns, information sources and exposure to climate-related content.

### Secondary school students’ following the news about climate change

The results indicate that the student cohort was approximately evenly divided in terms of their engagement with climate change news. Specifically, 18 students (48.6%) actively followed such news, whereas 19 students (51.3%) did not. Among those who engaged with the news, females were predominant, comprising 15 females and three males. Conversely, the non-follower group consisted of ten males and eight females.

The findings revealed a moderate level of interest, with slight variations attributable to gender, highlighting the necessity of integrating gender-sensitive strategies into environmental education.

### Secondary school students’ reading status of books on climate change

Only 2.7% of the students, represented by a single individual, indicated that they had read a book on climate change, specifically “Global Warming” published by TÜBİTAK. In contrast, 97.3% of the students (36 individuals) reported not having engaged with any literature on the topic. The findings indicate that students possess a limited comprehension of climate-related issues through formal reading materials, underscoring a deficiency in accessible environmental literature appropriate for their age group.

### Sources used by students to access climate change information

Students reported utilizing a diverse array of sources to obtain information on climate change, with Google being the most frequently referenced source, as cited by 13 students (35%). Furthermore, 10 students (27%) reported using both Google and social media, whereas six students (16%) relied exclusively on social media. Additionally, seven students (18%) indicated that they employed both television and Google, and one student (2%) identified the school as their primary source of information. The predominance of digital platforms over traditional educational methods underscores the need for a more systematic integration of climate education into school curricula.

### Frequency of students’ encountering news related to climate change

Approximately 48% of students reported encountering climate-related news weekly, while 32% did so monthly and 18% annually. The findings indicate a relatively high level of engagement with climate-related media, particularly among students who utilize online platforms. However, this engagement does not necessarily correlate with reading habits or school-based learning.

#### Theme 1: conception

This section explores middle school students’ comprehension of climate change through their self-generated definition of it. The analysis revealed six interconnected interpretative frameworks rather than distinct categories. These frameworks demonstrate a spectrum of scientific understanding, experiential reasoning and conceptual misconceptions.

As illustrated in Table 1, the analysis revealed the emergence of six interconnected interpretative frameworks rather than independent categories.

**TABLE 1:** Students’ views on climate change perceptions.

Theme 1	Sub theme	Sample comments
Definitions of climate change	Climate change caused by an increase in greenhouse gases	Increases in greenhouse gases, changes in the average state or variability of the climate over decades or longer, regardless of the cause of climate change. (S18)
		Climate changes with the increase of greenhouse gases that retain heat in the atmosphere. (S27)
		Climate change caused by the increase of greenhouse gases that retain heat in the atmosphere. (S30)
	Climate change caused by gases released into the atmosphere by humans	Climate change caused by the proliferation of toxic gases in the atmosphere. (S2)
		It is called the increase in the temperature of the earth as a result of the greenhouse effect of the gases given to the atmosphere by human beings. (S7)
		It is the negative impact of people on nature by using objects, tools, etc. that are against nature. It is the increase in temperature. (S21)
	Climate change caused by human and natural factors	It is called climate change caused by human, that is, human and environmental factors. (S1)
		It is a change caused by human influence in addition to natural changes over the long geological history of the Earth. (S14)
	Seasonal climate change	It is the change in climate with increasing temperature due to negative effects caused by human and environmental factors. (24)
		It means moving from winter to summer, summer to winter. (S5)
		To change from cold weather to hot weather. (S9)
	Weather events are experienced out of season	Variability of seasons and change of weather conditions. (S25)
		For example, experiencing summer in spring. (S6)
		Climates change at a moment’s notice, for example, it snows in summer. (S12)
		Global warming, i.e., winter conditions in summer and summer conditions in winter. (S28)
	Understanding what needs and wants are is an essential part of interpreting climate-related attitudes	Global warming affects the climate system of the world. (S4)
		Realization of unexpected events as a result of an increase in average temperature. (S32)
		Earth’s average temperatures are rising more than expected. (S37)

### Scientific explanations grounded in greenhouse gases

A cohort of students demonstrated a relatively advanced understanding of scientific concepts by identifying greenhouse gases as the primary contributors to climate change. Their explanations referenced the accumulation of gases that trap heat in the atmosphere and linked this phenomenon to long-term climate alterations. For example, one student characterized climate change as “a change in the Earth’s climate lasting for decades due to rising greenhouse gases.” These responses suggest the need to develop climate literacy and exposure to scientific discourse, potentially through media or classroom interactions.

### Human-induced environmental change

Numerous students identified anthropogenic activities as the primary drivers of climate change, citing pollution, industrial emissions, and the unsustainable use of resources as significant contributing factors. These individuals frequently employ moral or ethical language, underscoring human responsibility by characterizing it as “the outcome of people’s detrimental effects on nature.” This perspective reflects increasing environmental awareness shaped by personal values and societal narratives.

### Combined human and natural causes

A smaller subset of responses offered hybrid explanations, recognizing both natural and anthropogenic factors. These responses indicate a transitional phase in conceptual understanding, wherein the students endeavored to integrate diverse causal perspectives. This dual attribution mirrors the broader public discourse and may represent an evolving comprehension of the complexity inherent in climate systems.

### Misconceptions: climate change as seasonal variation

Many students erroneously equate climate change with typical seasonal transitions such as the shift from winter to summer. This prevalent misconception suggests that students often interpret the term “change” literally, relying on their everyday weather experiences rather than comprehending the intricate climate system processes. These misunderstandings underscore the necessity of explicitly teaching the distinction between weather and climate in educational curricula.

### Experiential interpretations: unseasonal weather events

Some students perceive climate change through anomalous weather events they have personally experienced, such as summer snowfall or unusually warm spring seasons. These perceptions exemplify experiential learning, wherein direct observation exerts a more significant influence on comprehension than abstract scientific concepts do. While these interpretations are intuitive, they underscore the challenges of correlating individual experiences with global climate patterns.

### Global warming and climate change as interchangeable concepts

Some participants primarily associated climate change with global warming, citing rising temperatures and disruptions to Earth’s equilibrium. Although these students recognized the global scope of the issue, their descriptions revealed difficulty in differentiating between these two interconnected yet distinct concepts.

#### Theme 2: daily life reflection

This section examines the third research question: How do middle school students perceive the impact of climate change on their daily lives? The analysis revealed that students understand climate change as a phenomenon that directly influences both natural systems and their everyday experiences. Rather than perceiving these impacts as isolated events, students described them as interconnected environmental, agricultural, and socioemotional outcomes. Seven related interpretive themes were identified in the study.

As illustrated in Table 2, the qualitative analysis suggests that students perceive climate change as having an increasingly disruptive influence on both natural systems and their lives. Rather than viewing these impacts as distinct, students described them as interconnected, influencing the environment, agriculture, and their socioemotional wellbeing. Seven interpretive patterns were identified.

**TABLE 2:** Students’ views on the effects of climate change on their daily lives.

Theme 2	Sub theme	Sample comments
Impact of climate change on daily life	Increase in natural disasters	It has a bad impact. Drought and thirst occur. We become homeless because of natural disasters. (S2)
		The crops in the field are affected because the weather is constantly deteriorating. Rainfall, storms and droughts can occur. (S19)
		Crop cultivation decreases and the balance is disturbed. When the seasons are unbalanced, diseases increase. Drought increases. (S31)
	Negatively affecting	Drought and flooding. (S3)
		Seasons can disappear, life can end. (S12)
		No one will be able to leave their homes because they cannot predict the weather at any time. (S24)
	Rise of the sea surface due to melting of glaciers	It got very hot and the poles started to melt. (S17)
	Water shortages	It affects badly. Because drought occurs and people get thirsty. (S30)
		We are engaged in agriculture, crops do not grow without rainfall. Our lakes and rivers are drying up. Therefore, the fish die. (S34)
	Negatively affects our health	It affects us badly. We can get sick when the climate changes frequently. (S13)
	Death of plants and animals	Many plants and animals are becoming extinct. (S1)
		Bad effects animals die, we can’t get fresh air. (S9)
		Affects animals and plants, oxygen depletion. (S11)
	The seasons are not on time	Summer comes later, winters are milder. (S14)
		Shifting seasons, causing droughts, floods in some regions. (S23)

### Disruption of seasonal rhythms and environmental predictability

Many students identified the most prominent effect of climate change in their lives as irregular seasonal patterns. They noted that summers seem to be delayed, winters are less severe, and weather changes occur unexpectedly. These observations suggest that climate instability is perceived as a disruption of environmental predictability and daily life, resulting in uncertainty regarding the natural order and timing.

### Rising frequency of natural disasters

Students frequently report that natural disasters, including storms, droughts and floods, are increasing in severity and frequency. These observations indicate heightened awareness of climate vulnerability. Students associate these events with actual or perceived threats, such as the potential loss of their homes, crop damage, and diminished safety, thereby underscoring the emotional impact of climate-related hazards on their lives.

### Agricultural and food-related concerns

Individuals, particularly those residing in rural regions, associate climate change with agricultural instability and with food scarcity. They elucidated how diminished rainfall and erratic seasonal patterns adversely affect crop production and livestock survival. This observation underscores the importance of a localized ecological understanding, suggesting that children perceive global climate change through the lens of their community’s livelihood and daily sustenance.

### Water scarcity and resource stress

Another frequently cited consequence is the water scarcity. Students observed that rivers and lakes are depleting, leading to a shortage of clean water for humans, animals, and agriculture. These observations underscore an increasing systems-level understanding of ecological interdependence, recognizing that water shortages affect not only human wellbeing but also overall environmental equilibrium.

### Global phenomena: melting ice and rising sea levels

Some students broadened their understanding to encompass global impacts, citing phenomena such as glacier melting and rising sea levels. Although these occurrences were not directly observable in their immediate environment, students appeared to rely on information from media or educational sources, demonstrating an awareness that local changes are interconnected with global processes.

### Health impacts and emotional reactions

Numerous students have associated climate change with both physical and emotional discomfort, observing an increase in illness during sudden weather changes. Their observations reflect a concrete understanding of the effects of climate change, linking fluctuating temperatures and environmental unpredictability to their health. This perspective underscores the human dimension of climate change as an experience directly encountered rather than an abstract concept.

### Threats to ecosystems and biodiversity

Ultimately, several students expressed concerns regarding the decline of plant and animal species, emphasizing issues such as extinction and the reduction of oxygen levels in the atmosphere. These empathetic perspectives demonstrate an initial comprehension of ecological challenges and moral consideration for non-human life, which aligns with the broader objective of promoting environmental responsibility among young individuals.

#### Theme 3: cause of climate change

This theme explored secondary school students’ perceptions of climate change causes. The participants predominantly attributed climate change to anthropogenic activities, reflecting their growing awareness of environmental responsibility. Their perspectives were categorized into three interrelated areas: greenhouse gas emissions, inadvertent consumption of natural resources, and the broader issue of global warming.

In Table 3, the fourth theme explores students’ perspectives on climate change origins. Most students attributed this to anthropogenic activities, particularly industrialization and consumption. Their responses demonstrated developing environmental literacy that integrated scientific reasoning, media awareness, and ethical considerations. Three interconnected categories were identified in this study.

**TABLE 3:** Students’ views on the causes of climate change.

Theme 3	Sub theme	Sample comments
**Causes of climate change**	Increasing greenhouse gases	Factors such as greenhouse gases, fossil fuel use, perfume and deodorant use have an impact. (S1)
		Increase in carbon dioxide, destruction of amazon forests, underutilization of renewable energy sources. (S16)
		People use deodorant, individual means. Leaving water and lamps on, not installing filters on factory chimneys. (S21)
		Greenhouse gases, methane gases, carbon dioxide, population growth, deodorant use, litter. (S32)
	Unconscious use of nature	Excessive use of fossil fuels and increased industrialization. (S35)
		Melting glaciers, deforestation, toxic waste in water, exhaust gases, greenhouse gases. (S2)
		Because people use nature unconsciously. (S4)
		Bad gases released into the atmosphere, rapid industrialization, pollution, waste, human factor, unconscious consumption habits, rapid destruction of natural resources. (S37)
	Global warming	Air pollution, global warming, decreasing amount of oxygen, water scarcity, melting and thawing of sea ice. (S11)
		Increasing desertification and floods, decreasing vegetation, not being able to observe the sky clearly. (S22)
		Melting glaciers and deforestation. (S27)

### Greenhouse gas emissions as the primary driver

Most students identified the increase in greenhouse gases, particularly carbon dioxide and methane, as the principal factor driving climate change in the UAE. They attributed these emissions to industrial activities, fossil fuel combustion, and routine human behavior. Furthermore, numerous participants emphasized deforestation and inadequate adoption of renewable energy sources. Some responses integrated scientific reasoning with media-influenced perspectives, associating climate change with activities such as “factory emissions, vehicle exhaust, and excessive energy use at home.” Others underscored personal responsibility, noting that “wasting electricity or leaving lights on contributes to pollution and harms the environment.”

While these explanations demonstrate a general understanding of the relationship between human activities and atmospheric changes, certain misconceptions persist, such as the belief that deodorant sprays are significant contributors to air pollution. This underscores the necessity of improving students’ scientific literacy, enabling them to distinguish more effectively between local pollution issues and global climate change processes.

### Unconscious use of natural resources and environmental degradation

Fewer students have examined the causes of climate change from a moral or ethical perspective, attributing it to human negligence of the environment. They identified deforestation, pollution, and excessive consumption as instances of “individuals exploiting nature without awareness” and “neglecting to protect what the Earth provides.” This viewpoint indicates a growing recognition of ecological ethics among students, where climate change is perceived not merely as a physical phenomenon but also as a reflection of human values and behavior.

### Global warming as a consequence and a cause

Some students employed the terms “global warming” and “climate change” interchangeably, suggesting an incomplete understanding of these concepts. They characterize global warming as “the Earth getting hotter and the poles melting,” which subsequently “changes everything.” These observations indicate that while students recognize rising temperatures as a significant issue, they occasionally perceive it as both a cause and effect of climate change. This misunderstanding suggests that educational strategies should emphasize climate change as a complex, systemic process influenced by multiple interacting factors rather than as a singular event.

### Secondary school students’ views on the main problems that may arise due to global warming

The analytical theme in Table 4 centers on students’ comprehension of the potential effects of global warming. Their responses exhibited varying degrees of environmental reasoning, ranging from basic observations to more sophisticated ecological considerations. Six principal interpretive categories were identified, which underscore both the direct environmental impacts and the broader societal implications.

**TABLE 4:** Secondary school students’ views on problems that may arise with climate change.

Views	*F*
Events resulting from the melting of glaciers	S1, S4, S5, S6, S9, S10, S12, S13, S16, S20, S23, S26, S36
Drought	S2, S7, S11, S21, S25, S27, S30, S31, S35
Extinction of plant and animal species	S8, S32, S33, S34, S37
Natural disasters	S14, S15, S19, S22, S24, S28
Increase in diseases	S17
Environment and air pollution	S3, S18, S29

### Melting glaciers and sea-level rise: a shared concern

Numerous students identified the melting of glaciers and rise in sea levels as significant global consequences of climate change. Their observations are frequently informed by educational experiences and media exposure, linking melting ice to flooding, habitat loss, and threats to coastal regions. Some students associated these phenomena with endangered species, such as polar bears, demonstrating an understanding of how global changes affect ecosystems beyond their immediate surroundings.

### Drought and water scarcity: a daily reality

Students from agricultural regions identified drought and water shortages as critical concerns. They expressed concerns regarding the desiccation of rivers and lakes, crop failures, and food scarcity. Their accounts demonstrated an ability to associate global warming with local economic challenges, indicating practical understanding of environmental risks.

### Loss of biodiversity: a moral and ecological concern

Numerous students expressed considerable concerns regarding the extinction of plant and animal species. Their comments reflected a compassionate and ecocentric viewpoint, perceiving the decline in biodiversity not merely as an environmental issue but as a moral obligation that involves human responsibility toward nature.

### natural disasters and environmental instability

Several participants observed an increasing frequency of natural disasters such as floods, storms, and heatwaves. Their narratives reflected an awareness of environmental instability, although they occasionally encountered difficulties in articulating causal relationships between climatic factors. Nonetheless, they demonstrated an intuitive understanding of the interconnections between rising temperatures, extreme weather events, and environmental risks.

### Health problems and disease

Few students associated global warming with health risks, such as the spread of diseases or heat-induced illnesses. This observation indicates that while students are cognizant of observable environmental changes, they possess a limited understanding of the indirect health implications of climate change, an area that could be further developed in educational curricula.

### Environmental and air pollution

Some students associated climate change with general pollution, characterizing the environment as increasingly “dirtier” and observing a deterioration in air quality. Although these observations may lack scientific precision, they reflect environmental concerns and a comprehensive perspective on ecological degradation, wherein various environmental issues are perceived as interconnected.

#### Theme 4: activities for prevention

### Secondary school students’ suggestions to reduce the rate of climate change

Upon querying the secondary school students participating in the study regarding their recommendations for addressing climate change, seven distinct categories were identified.

Table 5 elucidates students’ ecological awareness and environmental responsibility by examining their perspectives on climate change. The feedback was categorized into seven interpretive themes, ranging from personal lifestyle modifications to broader systemic recommendations. Some responses reflected environmental slogans, while others exhibited an understanding of sustainability concepts, such as reducing carbon footprints and responsible consumption.

**TABLE 5:** Students’ views on reducing the rate of climate change.

Theme 4	Sub theme	Sample comments
Ways to mitigate climate change	Using public transport	He drives less and does not pollute the environment. (S5)
		Using public transport and recycling products. (S24)
		We should use public transport. (S32)
	Utilization of renewable energy sources	We can use renewable energy sources. We can use environmentally friendly vehicles. We should consume less meat. (S2)
		Renewable energy sources should be used. The seas should not be polluted, filters should be installed in factory chimneys. (S20)
		We should use natural resources consciously. We should prefer recyclable products. (S25)
	Avoiding excessive consumption	We can reduce our ecological footprint by reducing consumption. (S10)
		Everyone should control themselves and reduce their own needs. (S31)
		We should save money, we should not consume natural resources unconsciously. We should not pollute the water. (S33)
	Use of local products	We should shop at local markets. (S11)
	Reducing plastic use	We should reduce plastic waste and use less water. (S14)
		We should reduce the use of bags and support recycling. (S23)
	Encouraging recycling Not having knowledge	Encouraging the use of recyclable bottles, bags, glass, etc. (S19)
		They can recycle and make advertisements on billboards encouraging recycling. (S28)
		I have no information. (S35)
		I don’t know. (S36)

### Promoting environmentally friendly transportation

Many students advocated for the use of public transportation or walking as alternatives to private vehicle use to mitigate pollution and carbon emissions. These recommendations reflect students’ awareness of the transportation sector’s contribution to greenhouse gas emissions and their recognition of shared mobility as an effective strategy for addressing climate change.

### Supporting renewable energy and conscious resource use

Numerous students have advocated for the implementation of renewable energy sources such as solar and wind power. Their feedback also emphasized the significance of conserving natural resources and mitigating pollution, with frequent references to factory emissions and waste management practices. This reflects the increasing awareness of the need for comprehensive solutions that extend beyond individual efforts.

### Avoiding excessive consumption

One of the most frequently proposed strategies is to reduce excessive consumption. Students emphasized the significance of moderation, self-regulation, and conscientious utilization of resources such as water and electricity. Phrases such as “managing our needs” or “avoiding unnecessary consumption” illustrate how sustainability has been adopted as a personal value.

### Using local products

A smaller subset highlighted the importance of supporting local production and consumption of food. These responses suggest that the students were beginning to comprehend the environmental impact associated with the transportation and packaging of imported goods.

### Reducing plastic waste

Students frequently expressed concerns about plastic pollution, particularly regarding single-use items such as bags and bottles. They associated the reduction of plastic usage with environmental cleanliness and the protection of marine ecosystems, thereby demonstrating an awareness of waste-related issues that are commonly emphasized in environmental campaigns.

### Encouraging recycling and environmental campaigns

Numerous participants identified recycling as an essential strategy for reducing waste and resource conservation. Some participants suggested initiatives such as advertisements or public messages to encourage community engagement in recycling efforts. This approach reflects not only an understanding of the issue but also a commitment to fostering collective and civic participation.

### Limited knowledge and uncertainty

While a minority of students reported insufficient knowledge of strategies to address climate change, this indicates that, despite widespread environmental awareness, there are still deficiencies in understanding. These gaps are particularly evident in the translation of concerns into informed and practical actions.

## Discussion

This study investigated secondary school students’ comprehension, perspectives, and reflections on climate change within the framework of sustainability education. The findings, derived from semi-structured interviews, were classified into five domains: level of interest, conceptual understanding, reflections in daily life, perceived causes of bullying, and suggestions for prevention. This section contextualizes these findings within international research, emphasizing Türkiye’s unique sociocultural environment and critically engaging with existing theoretical frameworks.

### Level of interest: cultural positioning and media dependency

Students’ limited engagement with climate change issues mirrors global patterns, albeit with distinct regional characteristics. In countries such as Australia and the United States, young people frequently engage with climate-related matters through digital activism rather than formal educational channels (Salguero et al., 2024). Similarly, Turkish students predominantly rely on social media for information. In contrast to their peers in highly industrialized and educationally advanced nations, where structured environmental curricula are more prevalent, students in Türkiye encounter an educational system in which climate education is not prioritized. This dependence on digital media, as emphasized by Stoddart et al. (2024), highlights the critical importance of media and information literacy in enabling students to evaluate online content critically and assess its scientific validity.

### Conceptualization: integrating local and global understandings

Students’ interpretations of climate change range from scientifically accurate descriptions to misconceptions that confuse climate change with seasonal variations. This combination of correct and incorrect reasoning reflects global research highlighting similar conceptual conflicts among young people (e.g., Kresin et al., 2025). However, Türkiye’s educational context, which integrates Western scientific perspectives with traditional ecological insights, provides a unique framework for examining this dynamic relationship. From a constructivist perspective (Ameel, 2021), students’ efforts to synthesize textbook information with their observations of local climate conditions demonstrate an active process of knowledge construction, consistent with the sociocultural theory of learning that emphasizes meaning-making within the context.

### Reflections on daily life: a cross-cultural perspective on localized climate awareness

Students frequently associate climate change with tangible and everyday phenomena such as droughts, declining agricultural productivity, and unpredictable weather patterns. This observation is consistent with research conducted in Southern Europe and Latin America, where young individuals also expressed concerns regarding local water supply and food security (Escoz-Roldán et al., 2020). In contrast to Northern European adolescents, who often perceive climate change as a global ethical issue, Turkish students emphasize practical, location-specific aspects. Their perspectives underscore that local environmental experiences shape climate awareness (Yemini et al., 2023). Given Türkiye’s strategic position at the intersection of Europe and Asia, integrating local environmental conditions with global sustainability themes could offer a valuable approach to enhancing climate education in diverse cultural and socio-economic contexts.

### Perceived causes: from moral responsibility to systemic awareness

Research indicates that students frequently associate climate change with anthropogenic activities, as evidenced by studies conducted in Europe and East Asia (Ma and Harris, 2025; Nyberg and Wright, 2025). Their emphasis on minor personal actions, such as reducing waste or avoiding aerosol products, reflects a focus on individual responsibility rather than structural factors. This phenomenon, termed “behavioral simplification” (Boca and Saraçlı, 2019), arises from environmental education that prioritizes personal ethics over systemic analyses. Engaging with ecological modernization theory and critical pedagogy may facilitate students’ understanding of the interconnections between industrial systems, policies, and collective efforts, thereby enabling a shift from personal environmentalism to transformative sustainability citizenship.

### Prevention and educational implications: toward critical and systems thinking

Students’ proposals, such as reducing consumption, recycling, or utilizing renewable energy, reflect increasing awareness of environmental responsibility (Prada da Silva et al., 2025). However, they predominantly emphasized individual actions. In contrast, research conducted in Germany and Canada indicates that students are increasingly engaging with issues such as climate justice and socio-political change (Stein, 2024). However, Turkish adolescents appear less involved in addressing systemic or policy-level issues. This discrepancy may be attributed to variations in civic culture and educational curricula in these countries. Integrating systems thinking (Tan et al., 2021) and critical sustainability education into Turkish schools could cultivate a more comprehensive understanding of the interconnections between energy policies, production systems, and collective governance in terms of environmental impact. Encouraging collaborative project-based learning, particularly through community partnerships, can transform passive awareness into active participation.

### Researcher reflexivity and methodological considerations

This study relied solely on interviews, capturing subjective perspectives rather than directly observing classroom interactions. The lack of triangulation through observations or student artifacts limits the depth of interpretation (Dahal, 2025). Additionally, the researcher’s background in environmental education may have subtly influenced the data interpretation. To address these concerns, researcher reflexivity was maintained through iterative coding and validation by peers (Wilson, 2025).

This study contributes to the global literature on climate education by situating its findings within a cross-cultural framework, offering insights from a rarely examined perspective. Türkiye’s position as a cultural and geographical nexus underscores the significant influence of local experiences, ethical values, and access to formal education on students’ perceptions of climate change in this country. While the findings align with global trends indicating limited systemic awareness, they also underscore the potential for integrating culturally relevant, reflective, and critically informed sustainability education into teacher education programs. Enhancing media literacy, promoting context-specific learning, and incorporating systems thinking into school curricula can collectively empower young individuals to understand climate change and become informed, locally engaged, and globally conscious citizens.

## Conclusion

This study investigated the perceptions, comprehension, and misconceptions of climate change among secondary school students in the broader context of sustainability education. The findings revealed that while students demonstrated increasing awareness of environmental issues, their understanding of the concepts was fragmented and frequently conflated with weather or seasonal changes. Their significant reliance on digital media as a primary information source has resulted in both heightened engagement and the dissemination of misinformation, highlighting the dual role of online platforms as educational resources and sources of distortion.

This study, situated in Türkiye—a region characterized by its unique cultural and geographical position at the intersection of Europe and Asia—offers significant insights into a non-Western educational context. In contrast to research conducted in Western industrialized countries, where students frequently perceive climate change through the lens of global justice, participants in this study predominantly understood it through local and tangible experiences, such as drought and agricultural challenges. This localized comprehension underscores the potential of place-based, culturally responsive methodologies to enhance climate literacy across diverse educational systems.

From an educational perspective, the findings underscore the significance of integrating climate change education across various disciplines using interdisciplinary, enquiry-based methodologies. Linking scientific concepts with students’ personal experiences can facilitate a more cohesive and meaningful understanding of the subject. The incorporation of digital media literacy is particularly crucial in assisting students in discerning credible information sources and avoiding misinformation. Furthermore, fostering systems thinking and critical sustainability education can motivate students to transition from individualistic perspectives to collective, action-oriented approaches to climate mitigation that are more effective. Educational institutions should also provide opportunities for student-led environmental initiatives and professional development programs for educators that promote reflective and transformative learning.

The methodological approach of the study, which utilized semi-structured interviews, limited its capacity to achieve triangulation. The researcher’s background in environmental education may have influenced data interpretation; however, strategies such as reflexivity and peer debriefing were employed to enhance the credibility of the study. Future research should employ mixed-methods approaches that incorporate interviews, classroom observations, and media analyses to provide a more comprehensive understanding of students’ climate literacy. Comparative studies across diverse cultural, geographical, and institutional contexts could further elucidate how these factors shape young people’s perceptions of and engagement with climate change.

In conclusion, the advancement of climate change education in Türkiye, as well as in other transitional contexts, necessitates a transition from mere knowledge dissemination to an approach that is critical, reflective, and action-oriented. It is imperative to empower students to connect global environmental issues with local contexts while fostering media literacy and systemic understanding to cultivate informed, responsible, and proactive citizens capable of contributing to a sustainable future.

## Data Availability

The raw data supporting the conclusions of this article will be made available by the authors, without undue reservation.
